# Safety and Efficacy of Rivaroxaban Versus Warfarin in Cerebral Venous Thrombosis: A Comprehensive Meta-Analysis

**DOI:** 10.3390/neurolint17110183

**Published:** 2025-11-08

**Authors:** Redoy Ranjan, Gie Ken-Dror

**Affiliations:** 1 Institute of Cardiovascular Research, Royal Holloway University of London, London TW20 0EX, UK; gie.kendror@rhul.ac.uk; 2 Department of Cardiac Surgery, Bangabandhu Sheikh Mujib Medical University, Dhaka 1000, Bangladesh

**Keywords:** rivaroxaban, warfarin, cerebral venous thrombosis, safety, efficacy

## Abstract

**Background:** Long-term management of cerebral venous thrombosis (CVT) with rivaroxaban is still under evaluation. The primary objective was to compare the contemporary evidence of the safety and efficacy of rivaroxaban versus warfarin in the long-term (≥ 6 months) treatment of CVT. **Methods:** We searched electronic databases up to 30 April 2025 for randomised control trials (RCTs) and observational studies in CVT management. We utilised the Mantel–Haenszel (M-H) method with a fixed-effects model to calculate risk differences (RDs) between rivaroxaban and warfarin arms. The ROB 2.0 and ROBINS-I tools were used to observe the risk of bias among included studies. **Results:** A total of 12 studies were identified (4 RCTs and 8 observational cohorts), evaluating 1174 patients treated with rivaroxaban (n = 262) or warfarin (n = 912). The rate of recurrence of venous thrombosis was 4% lower among rivaroxaban- compared to warfarin-treated patients (1.5% vs. 4.0%; RD = −0.04; *p* = 0.04). However, non-recanalisation events were identical among rivaroxaban and warfarin arms (16.4% vs. 16.5%; RD = −0.01, *p* = 0.68). Additionally, long-term all-cause mortality (*p* = 0.39), clinically relevant bleeding events (*p* = 0.18), new intracranial haemorrhage (*p* = 0.79), and good clinical outcome (*p* = 0.92) events were similar between rivaroxaban and warfarin arms. While RCTs and observational cohorts have methodological concerns and potential bias, we validated our results by excluding studies with serious or critical risks of bias to ensure the robustness of our findings. **Conclusions:** Compared to warfarin, rivaroxaban offers lower recurrence rates with similar efficacy and safety profiles along with improved clinical convenience.

## 1. Introduction

Cerebral venous thrombosis (CVT) is a rare cause of stroke, which accounts for ~0.5–1% of all strokes and requires prompt medical management to prevent significant neurological morbidity and mortality [1,2]. CVT leads to the obstruction of the cerebral venous sinuses, leading to intracranial hypertension and potential neurological sequelae. Despite receiving appropriate care, 6–10% of patients may experience severe and permanent disabilities, while about 15% may require bed rest or hospitalisation due to recurrent severe headaches resulting from CVT [3,4]. Although CVT can manifest across all age groups, its incidence is highest among middle-aged adults, particularly women, due to predispositional factors such as oral contraceptive use, pregnancy, and hypercoagulable states associated with puerperium [2,3,5]. A recent European study by Ranjan and colleagues [1] found that the average age of onset for men and women with CVT was 46 years and 37 years, respectively. Women with multiple risk factors developed CVT about 12 years earlier than those with no risk factors. Additionally, CVT can also occur in neonates and the elderly, often due to specific underlying causes such as genetic thrombophilia disorders, infections, malignancies, and autoimmune disorders [2,5,6]. Early and accurate diagnosis is imperative, given the nonspecific nature of CVT symptoms, which include headache, seizures, and focal neurological deficits [5,6,7]. Timely intervention mitigates morbidity and mortality risks. Advances in neuroimaging, particularly magnetic resonance imaging (MRI) and magnetic resonance venography (MRV), have significantly enhanced diagnostic accuracy, while therapeutic advancements have improved clinical outcomes [6,7,8,9]. Despite these improvements, CVT remains a rare cerebrovascular pathology, with an estimated incidence of ~1.5 cases per 100,000 person-years [2,10,11].

Long-term mortality associated with CVT depends on several factors, including age, underlying health conditions, and the effectiveness of treatment. Data from large-scale registries, such as the BEAST (Biorepository to Establish the Aetiology of Sinovenous Thrombosis) study [3,4], suggest that approximately 10% of CVT patients die within the first year of diagnosis, mainly due to recurrent thrombosis or systemic complications. Recent findings from the BEAST study [4] also confirmed that patients with advanced age, coma, seizures, or cerebral haemorrhage experience poor long-term prognoses. Additionally, people with CVT complicated by the involvement of multiple sinuses, systemic infections, and malignancies have worse prognoses than those presenting with isolated single venous sinus thrombosis [3,12,13]. The prognosis for CVT has significantly improved due to better clinical awareness, earlier detection through advanced imaging techniques, and the widespread adoption of anticoagulation therapy. While the initial clinical presentation can be severe, the majority of patients achieve favourable long-term outcomes with early diagnosis and anticoagulation therapy. Several studies report that ~85% of CVT patients attain a Modified Rankin Scale (mRS) score of 0–2, signifying functional independence following appropriate anticoagulation therapy [5,6,13]. Furthermore, complete neurological recovery (mRS 0–1) is documented in two-thirds of CVT cases. A key determinant of better long-term prognosis is the recanalisation of occluded cerebral veins, which occurs within months of initiating anticoagulation therapy in most CVT patients. Although recurrent CVT is relatively rare, the existing literature found an estimated annual incidence of 2–4%, further contributing to an overall favourable prognosis [2,5].

Existing European Stroke Organisation (ESO) and American Heart Association (AHA)/American Stroke Association (ASA) guidelines recommended using initial systemic heparinisation followed by long-term warfarin to prevent recurrence and improve clinical outcome [5,6]. However, maintaining a therapeutic international normalised ratio (INR) level with long-term warfarin therapy is challenging, with a significant risk of major bleeding and recurrence of thromboembolic events [14]. In contrast, studies have shown that rivaroxaban may be a potential alternative to warfarin due to better patient compliance and fewer adverse effects [15,16]. Approximately 40% of CVT patients have radiological evidence of intracranial haemorrhage at hospital admission, and it is ambiguous if the risk–benefit ratio of rivaroxaban can be anticipated in patients with CVT, like peripheral venous thromboembolism [15,16,17,18]. Although rivaroxaban was found to have a lower rate of intracranial bleeding events, it would be of great interest to physicians and CVT patients if an anticoagulant were found to have predictable effects and no need for therapeutic INR monitoring [14,17,18,19]. Moreover, since CVT is rare, a meta-analysis could validate the hypothesis by increasing the sample size rather than conducting randomised controlled trials (RCTs) with a large sample to compare the safety and efficacy of rivaroxaban and warfarin.

This meta-analysis aimed to determine the long-term anticoagulation safety and effectiveness of rivaroxaban and warfarin in patients with CVT.

## 2. Materials and Methods

**Figure 1:**
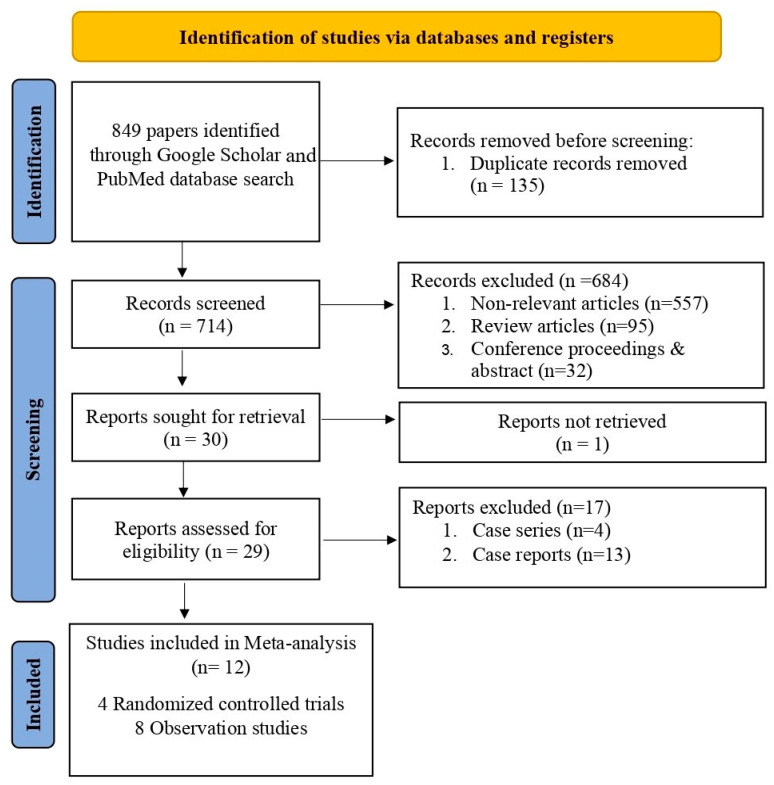
Flow diagram illustrating the different phases of our systematic review according to PRISMA 2020 guidelines [20].

This study followed the Preferred Reporting Items for Systematic Reviews and Meta-Analyses (PRISMA) 2020 guidelines [20] (Figure 1) and the Cochrane Handbook for Systematic Reviews of Interventions [21]. The study followed the PRISMA checklist, available in the Supplementary Materials (Table S1). This study was also registered with PROSPERO with an ID number CRD420251035152. We compared the effects of rivaroxaban and warfarin on long-term anticoagulation in CVT patients of all age groups, using both randomised control trials and observational cohorts [22,23,24,25,26,27,28,29,30,31,32,33]. The primary objective was to compare the efficacy of the recurrence and recanalisation events between the rivaroxaban and warfarin arms. The secondary endpoint was to compare the age difference and safety events represented by clinically relevant bleeding, new intracranial haemorrhage (ICH), good clinical outcome, and all-cause mortality between study groups.

Search strategy: We searched Google Scholar, PubMed, MEDLINE, EMBASE, CINAHL, and Web of Science Collection in all languages up to 30 April 2025 to assess rivaroxaban’s efficacy and safety against warfarin in treating CVT. We used Boolean terms and the search operators “AND” and “OR” to link search terms with search terms for disease, including (“cerebral venous thrombosis” OR “cerebral venous sinus thrombosis” OR “CVT” OR “CVST”) and search terms for treatment including (“rivaroxaban” OR “direct oral anticoagulant” OR “novel oral anticoagulant” OR “DOACs” OR “NOACs” OR “warfarin” OR “vitamin K antagonists” OR “factor Xa inhibitor”). We also used medical subject headings (MeSHs) for advanced literature searches, including thesis or dissertation repositories, preprint servers, and reference lists from preferred articles.

Study variables: We collected detailed methodological data on the included studies, particularly regarding the study type, population, and duration of treatment with rivaroxaban and warfarin. We compared age differences, recurrence of venous thrombosis and non-recanalisation events, clinically relevant bleeding (including both intracranial and extracranial bleeding), Modified Rankin Scale (mRS) scores of 0–2, and all-cause mortality as defined by the authors and previously published literature [34]. The mRS score is a 6-point scale ranging from 0 (no symptoms) to 6 (death), with a score of ≤2 indicating a good clinical outcome representing slight disability but the ability to look after their affairs without assistance [35]. Non-recanalisation events were defined as any case that failed to entirely or partially dissolve the thrombus following additional imaging. Recurrence of venous thrombosis was defined as the occurrence of a second event of CVT or any DVT despite long-term anticoagulation therapy.

Risk-of-bias analysis: This meta-analysis utilised the Cochrane Risk of Bias 2.0 (ROB 2) [36] and the Risk of Bias in Non-Randomised Studies—of Interventions (ROBINS-I) [37] tools to evaluate the risk of bias for each randomised trial and observational cohort, respectively. Two independent investigators performed a data search and risk-of-bias assessment to minimise errors, enhance reliability, and ensure an unbiased evaluation of the included studies’ quality. The RoB 2 tool’s evaluation covers five domains and is designed to assess the risk of bias in randomised controlled trial (RCT) studies. The five domains include bias due to the randomisation process, deviations from the intended interventions, missing outcome data, outcome measurement, and selectively reported results [36]. Each domain is assessed based on signalling questions that help determine whether the risk of bias is low, there are some concerns, or it is high. The first domain ensures that the randomisation process is truly random and well concealed. The second domain examines deviations due to a lack of blinding or improper intervention adherence. The third domain assesses whether missing data could bias the results, while the fourth looks at how outcome assessors might influence results through measurement errors or bias. The final domain evaluates whether selective reporting influenced the study’s findings. The responses to these questions guide an algorithmic judgement for each domain, which leads to an overall risk-of-bias judgement for the study, ensuring a transparent and systematic evaluation and reducing subjectivity in bias assessment. Furthermore, the ROBINS-I tool assesses bias across seven domains to evaluate the validity of non-randomised studies, observing various sources of systematic bias and aiding in determining the overall risk of bias in the study [37]. The seven domains covered by the ROBINS-I tools are study bias due to confounding, selection of participants, classification of interventions, deviations from intended interventions, missing data, measurement of outcomes, and selection of reported results. Each domain is assessed for potential bias based on study design and conduct and is then categorised as having low, moderate, serious, or critical risk bias based on pre-specified signalling questions. Confounding bias examines an imbalance in baseline characteristics, while selection bias considers how participants enter the study. Additionally, classification bias assesses the misclassification of interventions, while deviation bias looks at adherence to protocols. Furthermore, missing data bias measures loss to follow-up, and measurement and reporting biases evaluate assessment reliability and selective reporting. The overall risk of bias is determined by the highest risk level in any domain, which ensures a thorough evaluation of biases in non-randomised studies; for example, if a domain is rated as “critical,” the entire study is considered critically biased.

Statistical analysis: This study used RevMan v5.4 software to perform a meta-analysis. Risk differences, confidence intervals, and heterogeneity were calculated utilising the Cochran–Mantel–Haenszel method and a fixed-effects model. The study explored all sources of heterogeneity (clinical, methodological, and statistical) and observed inter-study heterogeneity (I^2^ index). The I^2^ statistic measures the percentage of total variation attributed to heterogeneity rather than random chance. We utilised the standard cut-off points, which are categorised as follows: low (0–25%), moderate (25–50%), substantial (50–75%), and high (>75%) heterogeneity. These indicate varying degrees of variability across studies. Statistical significance was considered if the *p*-value < 0.05.

## 3. Results

**Figure 2:**
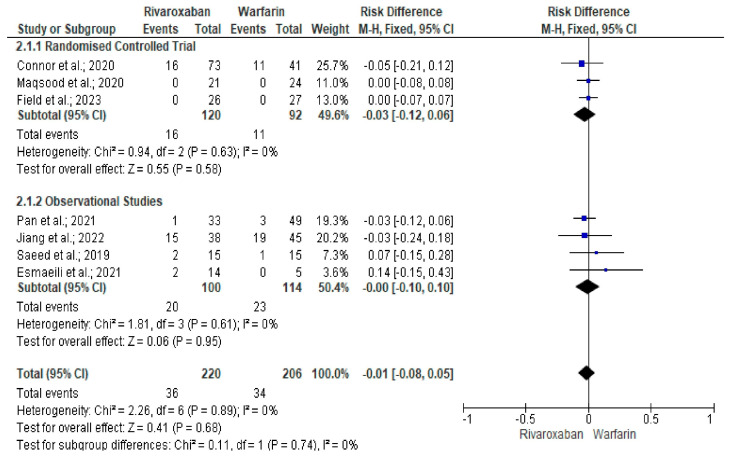
Forest plot comparing non-recanalisation outcome between rivaroxaban and warfarin arms [22,24,25,26,27,28,29].

**Figure 3:**
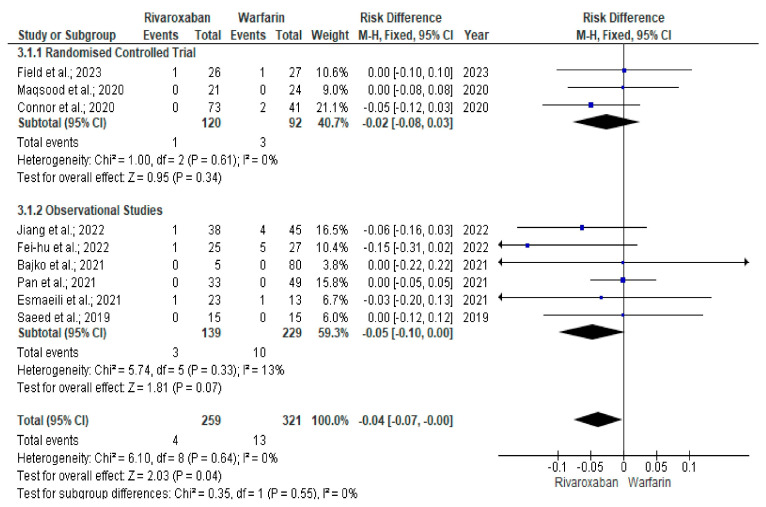
Forest plot comparing the recurrence of venous thrombosis between rivaroxaban and warfarin arms [22,24,25,26,27,28,29,30,32].

We identified a total of 12 studies [22,23,24,25,26,27,28,29,30,31,32,33] (4 RCTs and 8 observational studies) evaluating 1174 CVT patients (rivaroxaban arm, n = 262 and warfarin arm, n = 912) that met the study inclusion criteria (PRISMA flowchart) (Figure 1). The most common reasons for excluding articles were duplication (n = 135), incompatibility with the current study’s objectives (n = 557), conference proceedings (n = 32), case series (n = 4), and case reports (n = 13). We excluded duplicate studies to ensure each study uniquely contributed to the analysis, as well as those not aligned with our study objectives, for example, in their methodology and outcomes, to enhance the consistency and reliability of the findings. The clinical characteristics and study outcomes of the patients are shown in Table 1. There was no significant age difference (mean difference [MD] = 1.23, 95% CI −0.16–2.61, *p* = 0.08, I^2^ 0%) between the rivaroxaban and warfarin treatment arms in patients with CVT (Figure S1) [22,23,24,25,26,27,28,29,30,31,32,33]. Although non-recanalisation events (16.4% vs. 16.5%; RD = −0.01, 95% CI −0.08–0.05, *p* = 0.68, I^2^ 0%) were similar among study groups (Figure 2) [22,24,25,26,27,28,29], the rate of recurrence of venous thrombosis was significantly lower among rivaroxaban-treated patients than in the warfarin arms (1.5% vs. 4.0%; RD = −0.04, 95% CI −0.07–0.00; *p* = 0.04, I^2^ 0%) (Figure 3) [22,24,25,26,27,28,29,30,32].

**Figure 4:**
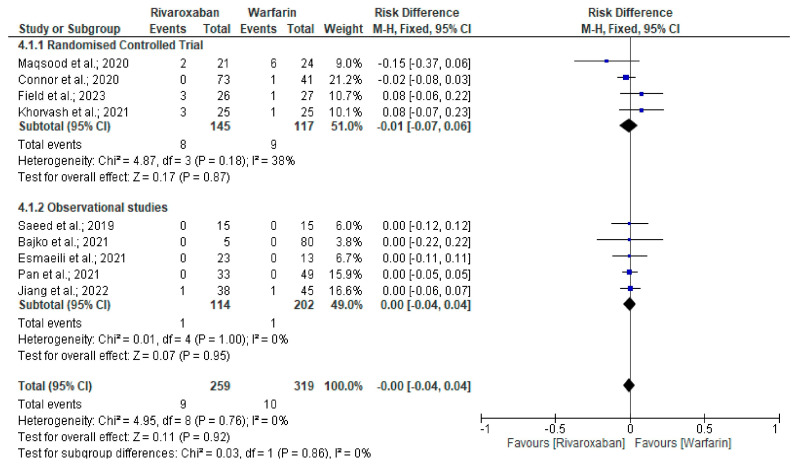
Forest plot comparing the clinically relevant bleeding events between rivaroxaban and warfarin arms [22,23,24,25,26,27,28,29,30].

**Figure 5:**
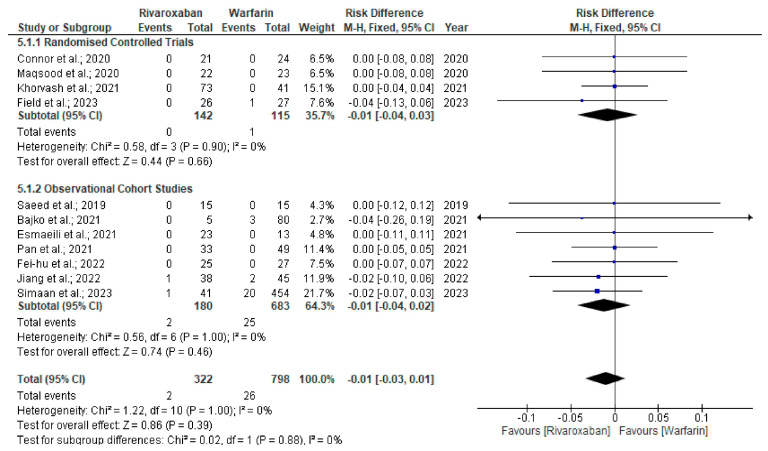
Forest plot comparing all-cause mortality between rivaroxaban and warfarin arms [22,23,24,25,26,27,28,29,30,31,32].

A good clinical outcome, determined as an mRS score of 0–2, was similar (94.5% vs. 85.6%, RD = −0.00, 95% CI −0.07–0.05, *p* = 0.92, I^2^ 0%) between the rivaroxaban and warfarin therapy arms (Figure S2) [22,23,26,27,28,29,30,31]. Furthermore, a comparison of safety events observed similar long-term effects in CVT patients in terms of clinically relevant bleeding (3.4% vs. 3.1%; RD = −0.00, 95% CI −0.04–0.04, *p* = 0.92, I^2^ 0%) (Figure 4) [22,23,24,25,26,27,28,29,30] and all-cause mortality (0.6% vs. 3.3%; RD = −0.01, 95% CI −0.03–0.01, *p* = 0.39, I^2^ 0%) (Figure 5) [22,23,24,25,26,27,28,29,30,31,32]. Additionally, the occurrence of new-onset ICH (1.1% vs. 1.2%; RD = −0.00, 95% CI −0.03–0.03, *p* = 0.79, I^2^ 0%) (Figure S3) [22,23,24,25,26,27,28,29,30,32] events was similar between the rivaroxaban and warfarin arms.

### Quality and Bias Assessment

**Figure 6:**
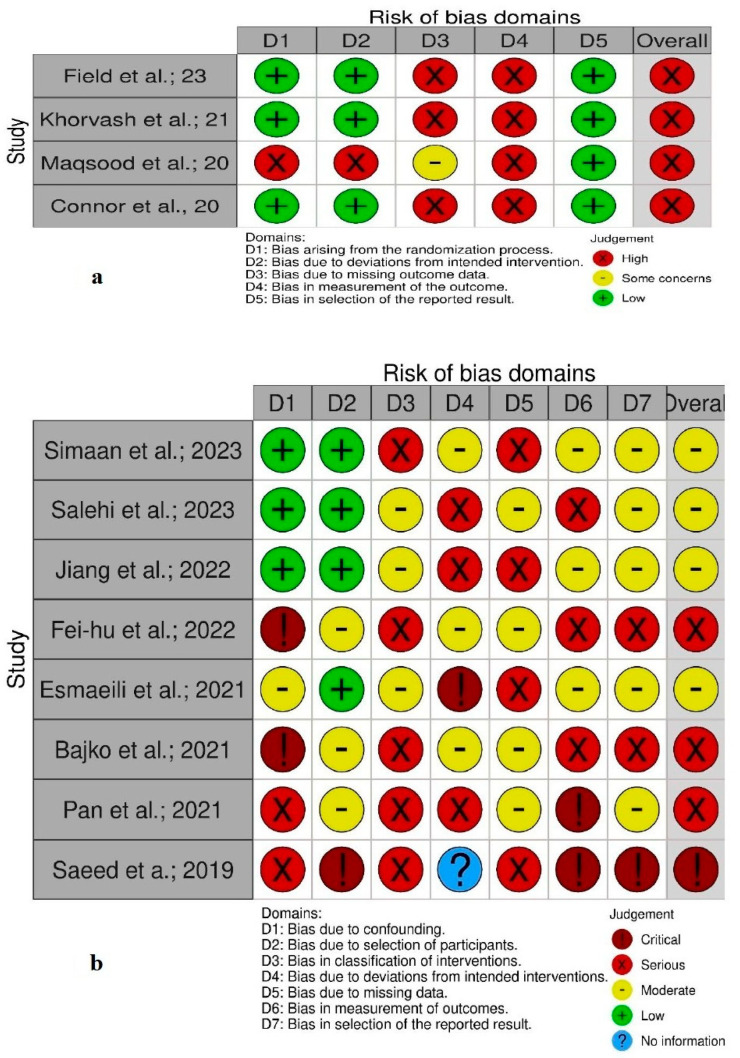
Risk-of-bias graph for RCTs (**a**) and observational cohort studies (**b**) [22,23,24,25,26,27,28,29,30,31,32,33].

All RCTs [22,23,24,25] had methodological concerns regarding the randomisation process, deviations from intended interventions, and bias in the outcome measurement and were judged to be at high risk of bias (Figure 6a). Moreover, all observational cohorts had at least moderate risk bias; three studies had a serious risk of bias [28,30,32], and one study [29] had a critical risk of bias (Figure 6b). Significant heterogeneity was observed only for non-recanalisation events between study groups, which was cross-validated by repeating the analysis and excluding the outlier study in this meta-analysis. To enhance the robustness of the study results and address identified biases, all analyses were repeated, excluding studies with a severe and critical risk of bias; however, the overall results remained unchanged. Additionally, the symmetrical funnel plot indicates that there is no evidence of publication bias. To obtain robust study results, we focused specifically on patients treated with rivaroxaban, whereas previous meta-analyses [34,38,39,40,41,42] analysed multiple DOAC agents compared to vitamin K antagonists, such as warfarin and phenprocoumon.

## 4. Discussion

Comparing long-term CVT outcomes between rivaroxaban and warfarin, we find a significantly lower recurrence of venous thrombosis in rivaroxaban-treated patients despite similar age groups. Further, we show that rivaroxaban-treated patients experienced comparable non-recanalisation, all-cause mortality, clinically relevant bleeding, new ICH, and good clinical outcomes based on an mRS score of 0–2.

Despite anticoagulation therapy having improved survival rates, long-term warfarin treatment poses challenges, including a higher risk of bleeding complications [14,43,44]. The annual incidence of major bleeding events in warfarin-treated CVT patients is estimated at 2–3%, with cumulative rates of 5–10% over long-term follow-up. Elderly individuals, patients with renal insufficiency, and those with a history of major bleeding events are particularly vulnerable to major bleeding events [14,45]. Maintaining international normalised ratio (INR) levels within the therapeutic range of 2 to 3 minimises bleeding risk while ensuring effective anticoagulation, specifically for recanalisation and preventing recurrence. A major concern with anticoagulation therapy in CVT patients is ICH, occurring in ~5% of long-term cases, especially with an INR level of >3.5, which is significantly higher in individuals with cerebral small-vessel disease, hypertension, or a history of haemorrhagic stroke [46,47]. Insofar as anticoagulation strategies are concerned, long-term anticoagulation should be individualised to balance thrombotic risk reduction with the risk of bleeding complications, and DOACs may be a safe alternative to warfarin due to their reduced bleeding risk and more predictable pharmacokinetics [45,46,47,48]. Nevertheless, from a clinical perspective, the predictable pharmacokinetic profile of rivaroxaban obviates the need for routine INR monitoring, thereby enhancing patient adherence and minimising dietary and drug interactions [19,49,50,51].

Recent clinical studies suggest that the recurrence of venous thrombosis following an initial CVT episode is relatively low compared to other forms of venous thromboembolism (VTE), such as deep vein thrombosis (DVT) and pulmonary embolism [52,53,54,55]. Long-term venous thrombosis recurrence rates in warfarin-treated CVT patients are ~7% per year, with cumulative recurrence rates of 5–15% over a 10-year follow-up period [56,57,58]. The CVT recurrence risk is higher in individuals with hereditary prothrombotic disorders, including factor V Leiden mutation, antiphospholipid syndrome, and deficiencies in protein C, protein S, or antithrombin [57,59,60]. Extended anticoagulation therapy for 6 to 12 months may be lifelong, especially in patients with hereditary thrombophilic conditions, which helps reduce the risk of recurrent thrombotic events [5,6,58]. Nonetheless, lifelong anticoagulation is often necessary for individuals with hereditary thrombophilia-related CVT due to a persistent hypercoagulable state that is associated with the risk of recurrent thrombosis [61,62]. These conditions disrupt the coagulation cascade, leading to a significantly higher recurrence rate of venous thromboembolism, which is why prolonged anticoagulation is necessary to prevent recurrence and promote recanalisation rates for better prognosis. However, decisions regarding lifelong anticoagulation therapy are individualised, considering factors like prior thrombotic history, family history, and associated risk factors to optimise clot prevention with the bleeding risk associated with prolonged anticoagulation in CVT [62,63,64]. Furthermore, a recent IRIS (Italian Registry in the setting of atrial fibrillation ablation with rivaroxaban) study [16] analysing the safety and efficacy of rivaroxaban at 12-month follow-up in patients undergoing catheter ablation for atrial fibrillation determined its primary safety and efficacy, as represented by the occurrence of all-cause mortality, systemic embolism, and major bleeding events, which also supports our study’s findings.

Unlike this meta-analysis, a previous meta-analysis [34,38,39,40,41,42] was based on multiple DOAC agents versus vitamin K antagonists (VKAs). Although current guidelines [5,6] recommend warfarin as a long-term CVT treatment, significant questions remain, e.g., risk of bleeding, regular follow-up with therapeutic international normalised ratio level, and interactions with food and drugs [14,65]. Recent meta-analyses indicate that DOACs and warfarin have similar long-term safety and efficacy profiles for managing CVT. In a recent, most up-to-date comprehensive meta-analysis evaluating 25 RCTs and observational studies involving 2301 CVT patients, Ranjan and colleagues [34] suggest that DOACs may be preferred due to their ease of clinical management over warfarin, supporting existing study findings. One of the limitations of this meta-analysis was that the authors included childhood CVT in their analysis, as the childhood CVT aetiology may differ from adult CVT; however, a study excluding child CVT in its analysis also observed no significant difference in overall study outcomes. Although their results were similar to those of existing meta-analyses, Ranjan and his colleagues’ [34] findings were more clinically significant and novel than those of previous meta-analyses because they excluded phenprocoumon-based studies; phenprocoumon is a vitamin K antagonist, whose pharmacodynamics differ from those of warfarin, which could introduce potential outcome bias. Additionally, Yaghi and colleagues [38] found that the risks of recurrent venous thromboembolism, major haemorrhage, intracranial haemorrhage, mortality, and complete venous recanalisation were similar between DOAC and vitamin K antagonist (warfarin and phenprocoumon) treatment arms. In another meta-analysis of 17 studies, Nepal and colleagues [39] demonstrated that DOACs have comparable safety and efficacy profiles to VKAs but show better recanalisation rates, supporting our current study’s findings. To the best of our knowledge, this is the first meta-analysis comparing rivaroxaban versus warfarin in the long-term management of CVT, and it has produced novel findings showing significantly lower recurrence of venous thrombosis and better recanalisation in rivaroxaban-treated patients despite similar age groups and safety profiles.

Warfarin-related haemorrhagic risk is affected by patient comorbidities, concurrent medication use (such as antiplatelet therapy), and dietary factors [66,67,68]. In the absence of sufficient powered evidence in CVT treatment outcomes, rivaroxaban, a commonly used DOAC, and warfarin are both utilised in the long-term treatment of DVT and pulmonary embolism, demonstrating comparable safety and efficacy outcomes [69,70,71,72]. Comparative clinical trials, including the EINSTEIN-DVT study [73], have evaluated their relative efficacy, safety, and practical implications in real-life scenarios. In terms of efficacy, rivaroxaban has demonstrated non-inferiority to warfarin in preventing recurrent VTE, with the EINSTEIN-DVT trial [73] reporting similar rates of recurrent DVT and PE between the two therapeutic agents, supporting the findings of other published articles [74,75]. However, rivaroxaban offers a fixed-dose regimen without necessitating initial parenteral anticoagulation, unlike warfarin, which requires bridging with heparin, and rivaroxaban is associated with a lower incidence of major bleeding, particularly intracranial haemorrhages, compared to warfarin. Additionally, rivaroxaban is becoming increasingly popular due to its early onset and offset of action, shorter plasma half-life, lack of need for regular dose adjustments, and fewer food and drug interactions, which supports our study findings [14,76,77,78]. The results from this meta-analysis find that improved efficacy supports the use of rivaroxaban as an alternative to warfarin for CVT treatment with similar long-term safety compared to warfarin.

As with any study, there are limitations to our work. Although observational cohorts are potential sources of bias, we cross-validated significant results, excluding papers with a serious risk of bias as well as a critical risk of bias, which should mitigate risk [79]. Considering the rare occurrence of CVT and the lack of large RCTs, current meta-analyses provide rational findings that shed light on the better efficacy of rivaroxaban as a long-term treatment for CVT compared to the current guideline [5,6]. This study utilised a fixed-effects model as the included studies were methodologically similar with overall low heterogeneity, allowing for a focused interpretation of the effect size and leading to greater statistical power and more precise estimates [80]. Most studies had treatment durations ranging from 3 to 12 months, with 6 months being the most common; hence, we extrapolate a 6-month follow-up as indicative of long-term outcomes, though variations in treatment duration may introduce outcome bias. Despite the diversity in long-term anticoagulation use, interstudy heterogeneity was insignificant except for non-recanalisation events, which were cross-validated utilising a random-effects model to mitigate the risk of outcome bias. Further, the aetiology of CVT in children and adults may differ [81,82]; our analysis excludes childhood CVT in concordance with the overall consensus. While the small sample size in RCTs, lack of events in some studies, and varying drug dosages and brands limit this analysis, it remains the most extensive analysis conducted to date. Despite the overly restrictive nature, we have utilised the fixed-effects model in our meta-analysis as the study events observed negligible (I^2^ 0%) heterogeneity, so we extrapolate that the true effect size is the same across all studies [83]. Nevertheless, heterogeneity thresholds are guidelines, not strict rules, so interpretations should consider study characteristics, effect sizes, and confidence intervals. Further, conducting a meta-analysis with few studies and small populations makes it more challenging to detect substantial heterogeneity, which is why a fixed-effects model is used to precisely estimate the common effect across studies [83,84]. Finally, a narrow CI may raise concerns; however, risk difference is an absolute measure, unlike relative risk or odds ratio, so small values are expected when event rates are low, especially when they are zero, and similar between study groups [84,85]. Additionally, fixed-effects models do not account for variability between studies, leading to narrower CIs due to lower variance in pooled estimates; that is why these models provide greater statistical precision in small event studies. Finally, given the limited sample size, which excluded analyses by therapy duration or risk of bias, we acknowledge this as a study limitation and recommend that future, large-scale investigations specifically evaluate long-term outcomes and duration-specific effects in CVT management.

## 5. Conclusions

Rivaroxaban-treated CVT patients had a significantly lower CVT recurrence rate than those treated with warfarin, despite an identical age group of the population. While good clinical outcomes, all-cause mortality, recanalisation, and clinically relevant bleeding were similar between treatment arms, rivaroxaban might be preferred over warfarin for long-term CVT management due to its similar safety and efficacy profiles and more convenient clinical use. However, further prospective studies and large-scale randomised controlled trials are necessary to confirm the robustness of our study findings and provide stronger evidence for its use in CVT management.

## Figures and Tables

**Table 1:** Basic methodological characteristics and study variables of studies included in the meta-analysis [22,23,24,25,26,27,28,29,30,31,32,33].

Sl Number	Authors, Year	Study Design	Location	Sample Size (N)	Age (years)	Female (%)	Treatment Duration (months)	Follow-Up (months)	Recurrence (n/N)	New ICH (n/N)	Clinically Relevant Bleeding (n/N)	Non-Recanalisation (n/N)	mRS 0–2 (n/N)	Mortality (n/N)
Randomised Controlled Trial														
**1**	Field et al.; [22]	RCTs	Canada	Rivaroxaban (26)	48.5 (37–64)	69.2%	6–12	6–12	1/26	2/26	3/26	0/26	22/23	0/26
				Warfarin (27)	48 (39–58)	63.0%	6–12	6–12	1/27	1/27	1/27	0/27	23/25	1/27
**2**	Khorvash et al.; [23]	RCTs	Iran	Rivaroxaban (25)	41.20 ± 11.4	68%	6	6	**-**	1/24	3/25	-	22/22	0/22
				Warfarin (25)	40.76 ± 11.7	76%	6	6	**-**	0/25	1/25	-	23/23	0/23
**3**	Maqsood et al.; [24]	RCTs	Pakistan	Rivaroxaban (21)	26 (15–36)	86%	3–12	12	0/21	0/21	2/21	0/21	-	0/21
				Warfarin (24)	27 (15–45)	79%	3–12	12	0/24	0/24	6/24	0/24	-	0/24
**4**	Connor et al.; [25]	RCTs	Multi-centre	Rivaroxaban (73)	≤17	37%	3	3	0/73	0/73	0/73	16/73	-	0/73
				Warfarin (41)	≤17	44%	3	3	2/41	1/41	1/41	11/41	-	0/41
Observational Studies														
**5**	Jiang et al.; [26]	Retrospective observational	China	Rivaroxaban (38)	38.9 ± 14.2	44.7%	3–12	3	1/38	0/38	1/38	15/38	38/38	1/38
				Warfarin (45)	40.9 ± 13.2	33.3%	3–7	3	4/45	1/45	1/45	19/45	45/45	2/45
**6**	Esmaeili et al.; [27]	Retrospective observational	Iran	Rivaroxaban (23)	36 ± 11.2	73.1%	12	12	1/23	0/23	0/23	2/14	22/22	0/23
				Warfarin (13)	34 ± 11.2	92.3%	12	12	1/13	0/13	0/13	0/5	10/10	0/13
**7**	Pan et al.; [28]	Prospective observational	China	Rivaroxaban (33)	37.1 ± 13.8	66.7%	6	6	0/33	0/33	0/33	1/33	33/33	0/33
				Warfarin (49)	34.1 ± 14.0	67.4%	6	6	0/49	0/49	0/49	3/49	48/49	0/49
**8**	Saeed et al.; [29]	Retrospective observational	Pakistan	Rivaroxaban (15)	31.47 ± 9.2	66.7%	6	6	0/15	0/15	0/15	2/15	15/15	0/15
				Warfarin (15)	35.87 ± 10.8	73.3%	6	6	0/15	0/15	0/15	1/15	15/15	0/15
**9**	Bajko et al.; [30]	Retrospective observational	Romania	Rivaroxaban (5)	54.2 ± 18.7	40%	13.2	13.2	0/5	0/5	0/5	-	4/5	0/5
				Warfarin (80)	40.3 ± 15.2	67.5%	6	6	0/80	0/80	0/80	-	77/80	3/80
**10**	Simaan et al.; [31]	Prospective	Israel	DOAC (41)	45.6 ± 19.8	66%	3	3	**-**	**-**	**-**	**-**	32/41	1/41
				Warfarin (454)	41.3 ± 18.3	67%	3	3	**-**	**-**	**-**	**-**	359/454	20/454
**11**	Fei-hu et al.; [32]	Prospective observational	China	Rivaroxaban (25)	35.80 ± 3.15	56%	3	-	1/25	0/25	1/25	**-**	**-**	0/25
				Warfarin (27)	34.37 ± 3.02	55.6%	3	-	5/27	1/27	8/27	**-**	**-**	0/27
**12**	Salehi et al.; [33]	Retrospective observational	USA	Rivaroxaban (6)	46.1 ± 17.4	67.4%	6	6	**-**	**-**	**-**	6/65	**-**	**-**
				Warfarin (43)	44.3 ± 16.1	63.0%	6	6	**-**	**-**	**-**	43/65	**-**	**-**

N = sample size; n = events; RCT—randomised controlled trial; ICH—intracerebral haemorrhage; mRS—Modified Rankin Scale. Age is reported as the mean ± SD or as the median (interquartile range), as specified in the original articles. Furthermore, the blank squares indicate that no data were available for those variables in that study.

## Data Availability

The data supporting the findings of this study are available within the article and its Supplementary Materials.

## Supplementary Materials

The following supporting information can be downloaded at https://www.mdpi.com/article/10.3390/neurolint17110183/s1, Figure S1: Forest plot comparing age differences between rivaroxaban and warfarin arms; Figure S2: Forest plot comparing good clinical outcomes based on mRS 0–2 score between rivaroxaban and warfarin group of population; Figure S3: Forest plot comparing the occurrence of new intracranial haemorrhage (ICH) between rivaroxaban and warfarin arms; Table S1: PRISMA checklist.

## Abbreviations

The following abbreviations are used in this manuscript:

CVT	Cerebral venous thrombosis
PRISMA	Preferred Reporting Items for Systematic Reviews and Meta-Analyses
ROB	Risk of bias
ROBINS-I	Risk of Bias in Non-Randomised Studies—of Interventions
